# Editorial: Crosstalk between thyroid hormones, analogs and ligands of integrin αvβ3 in health and disease. Who is talking now?

**DOI:** 10.3389/fendo.2022.1119952

**Published:** 2023-01-05

**Authors:** Sandra Incerpi, Osnat Ashur-Fabian, Paul J. Davis, Jens Z. Pedersen

**Affiliations:** ^1^ Department of Sciences, University Roma Tre, Rome, Italy; ^2^ Translational Oncology Laboratory, Meir Medical Center, Kfar-Saba, Israel; ^3^ Department of Human Molecular Genetics and Biochemistry, Sackler School of Medicine, Tel Aviv University, Tel Aviv, Israel; ^4^ Department of Medicine, Albany Medical College, Albany, NY, United States; ^5^ Pharmaceutical Research Institute, Albany College of Pharmacy and Health Sciences, Albany, NY, United States; ^6^ Department of Biology, University Tor Vergata, Rome, Italy

**Keywords:** integrin αvβ3, signal transduction, thyroid hormone receptor, nongenomic mechanism, cancer, COVID-19, Na/K-ATPase, tetraiodothyroacetic acid (tetrac)

The classical molecular mechanism of thyroid hormone action is genomic, involving nuclear thyroid hormone receptors (TRs) complexed with 3,5,3’-triiodo-L-thyronine (T_3_) and transcription of specific genes (1). A nongenomic pathway is now understood to exist and is particularly important to tumor cells (1, 2). The pathway begins at a cell surface thyroid hormone analogue receptor on plasma membrane integrin αvβ3; the principal hormone at physiological concentrations at this receptor is L-thyroxine (T_4_), the thyroid gland product that is the prohormone for T_3_. Downstream of the cell surface receptor, the hormone-integrin complex initiates intracellular signal transduction pathway sequences that culminate in differential expression of multiple genes relevant to cancer cell proliferation and tumor-linked angiogenesis (2). Certain of these signaling pathways can remarkably lead to TR activation in the nucleus. This issue of Frontiers in Endocrinology includes a set of papers focused on actions of T_4_ that are nongenomic in mechanism.

T_3_ is the principal ligand of nuclear TRs in all types of tissues, whereas T_4_, as noted above, is the major functional ligand for the cell surface αvβ3 integrin receptor for thyroid hormone analogues. The integrin is overexpressed and activated on cancer cells and rapidly-dividing endothelial cells. The specific cellular signal transduction pathways linked to the T_4_ receptor include mitogen-activated protein kinase (MAPK) and signal transducer and activator of transcription-1α (STAT-1 α) that lead to nuclear compartment co-activator and co-repressor proteins that regulate expression of specific genes related to tumor cell proliferation and tumor cell defense mechanisms. Among the proteins that move to the nuclear compartment as a result of the integrin-based actions of T_4_ include TRβ1, estrogen receptor- α (ER α) and tumor suppressor protein p53. The nuclear thyroid hormone receptor TRβ acts as an inhibitor of cancer cell proliferation and as a tumor suppressor.

TRα, instead, can be oncogenic as reported also in the first paper of the issue by Lasa and Contreras-Jurado, where the authors show the role of thyroid hormones in the inflammatory process leading to tumorigenesis and other pathological processes such as infectious disease up to Covid -19. The inflammatory process is described in details both in the short–term and in the chronic situation, as well as in the crosstalk between thyroid hormones and infection and thyroid hormones and cancer. A crosstalk between thyroid hormones and the immune system is widely recognized, thyroid hormones may behave as anti-inflammarory agents (3–6).

In the second paper, Ariyani et al. show that the TR-dependent signaling pathway *via* integrin αvβ3 plays a role in cerebellar development, neuritogenesis and dendritogenesis. The knockdown of integrin αv or integrin β3 or the double knock down of integrin αvβ3 significantly reduces dendritic arborization in Purkinje cells. The effect of thyroid hormones is mediated, in part, by activation of PI3-K and ERK1/2 (MAPK). The experiments were carried out mainly in primary culture of cerebellar Purkinje cells from mice and in cells in culture of neuroblastoma, Neuro –2A cells, with similar results, suggesting that neuritogenesis process is similar in primary culture and neuroblastoma cells.

The effects of thyroid hormones on tumor progression and metastasis is inhibited by tetraiodothyroacetic acid (tetrac) and nanoderivatives, tetrac is a metabolite of thyroid homone that binds the integrin αvβ3 at the RGD binding site or in close proximity to it (7, 8).

The paper of Tobi et al., shows by in-silico docking the affinity of more than 20 thyroid hormone metabolites with a variety of structural features sulfated, deiodinated, deaminated or decarboxylated, including the biologically active hormones T_3_ and T_4_, with integrin αvβ3. All the tested molecules show a negative energy suggesting good affinity with the RGD site, at the integrin, with differences. The RGD peptide was used both in linear or cyclic conformation, and it was found that iodination at the 3,5 and 3’ positions shows an increased affinity with respect to other iodination positions of other metabolites, but also the binding capability could be improved.

The Incerpi et al. paper is based on data on thyroid hormones, T_3_ and 3,5-diiodothyronine (3,5-T_2_) on the Na/K-ATPase in chick embryo hepatocytes. The metabolite 3,5-T_2_ shows a significant effect in the inhibition of the Na-pump, higher than T_3_, whereas T_4_ is without effect. The Na-pump is an ubiquitous enzyme present both in plant and in animal cells, whose inhibition by T_3_ and 3,5-T_2_ is mediated by PKA, PKC and PI3-K in the early stages of development. This inhibition gives rise to a protected or an anti-inflammatory environment, with high intracellular Na^+^ and Ca^2+^ ions, similar to the activation of the anti-inflammatory response elicited by the α7 acetylcholine receptor (α7nAChR) at the level of the vagus nerve (9). 3,5-T_2_ is a metabolite with strong effects on mitochondrial activity (resting metabolic rate) that is more efficient than T_3_, 3,5-T2 is also a vasodilator.

At the end of the story, we may find the answer to the question raised in the title of the Issue:

‘Crosstalk between thyroid hormones, analogs and ligands of integrin αvβ3 in health and disease. Who is talking now?’ The endogenous TH metabolites and their nanoderivatives may represent a therapeutic tool able to interact with integrin-modulated intracellular pathways in infective diseases and cancer. But we should also take into account the crossttalk between nongenomic and genomic signaling of thyroid hormones and steroid hormones and their analogues that appear to play a pivotal role in the physiopathology of cancer and in cancer management.

## Author contributions

PJD and SI wrote the first draft of the editorial. All the authors read, revised and approved the manuscript in the final form.
